# Grieves and struggles of family caregivers providing care for bedridden elderly patients affected by chronic degenerative diseases

**DOI:** 10.17533/udea.iee.v39n2e09

**Published:** 2021-06-15

**Authors:** Joice Soares Campos, Anna Cláudia Yokoyama dos Anjos, Sebastião Benício da Costa Neto, Rodrigo Sanches Peres

**Affiliations:** 1 MSc in Psychology. Psychologist at the Health Department of Araguari, Araguari-MG, Brazil. Email: joicepsicoufu@yahoo.com.br Health Department of Araguari AraguariMG Brazil joicepsicoufu@yahoo.com.br; 2 RN, PhD. Professor at the Federal University of Uberlândia, Uberlândia-MG, Brazil. Email: annaclaudia1971@gmail.com Universidade Federal de Uberlândia Federal University of Uberlândia UberlândiaMG Brazil; 3 Psychologist, PhD. Professor at the Pontifical Catholic University of Goiás, Goiânia-GO, Brazil. Email: sebastiaobenicio@gmail.com Pontifical Catholic University of Goiás GoiâniaGO Brazil sebastiaobenicio@gmail.com; 4 Psychologist, PhD. Professor at the Federal University of Uberlândia, Uberlândia-MG, Brazil. Email: rodrigosanchesperes@ufu.br. Corresponding author Universidade Federal de Uberlândia Federal University of Uberlândia UberlândiaMG Brazil rodrigosanchesperes@ufu.br

**Keywords:** caregivers, home nursing, aged, family health, qualitative research., cuidadores, atención domiciliaria de salud, anciano, salud de familia, investigación cualitativa., cuidadores, assistência domiciliar, idoso, saúde da família, pesquisa qualitativa.

## Abstract

**Objective.:**

To understand grieves and struggles of family caregivers providing care for bedridden elderly patients affected by chronic degenerative diseases.

**Methods.:**

This cross-sectional study was developed following the guidelines of the clinical-qualitative method. The sample was composed of 10 female family caregivers of bedridden elderly patients affected by chronic degenerative diseases in a city in the interior of Minas Gerais, Brazil. The sample size was determined by data saturation. The instruments used included semi-structured interviews and a field diary. The audio-recorded interviews were transcribed verbatim and submitted to content analysis. The field diary provided contributions to the organization of categories, conferring a more accurate context.

**Results.:**

The participants experienced two types of grief, one for the loss of a “healthy family member” and the other for the “announced death” of this person. Additionally, the participants faced two main struggles: overcoming (objective and subjective) fatigue and becoming fully capable of performing their roles.

**Conclusion.:**

The family caregivers of bedridden elderly patients affected by chronic degenerative diseases experience grieves and struggles that should be taken into account from the beginning of the care process through mental health actions intended to meet their needs.

## Resumen

**Objetivo.:**

Comprender los duelos y las luchas que experimentan cuidadores familiares de ancianos postrados en cama debido a enfermedades crónicas y degenerativas.

**Métodos.:**

El estudio tuvo un carácter transversal y se desarrolló de acuerdo con las directrices del método clínico-cualitativo. La muestra estuvo conformada por 10 cuidadoras familiares de ancianos postrados en cama debido a enfermedades crónicas y degenerativas de una ciudad del interior del Estado de Minas Gerais en Brasil. La muestra se cerró por saturación. Los instrumentos utilizados fueron un guión de entrevista semiestructurada y un diario de campo. Las transcripciones literales y textuales de las grabaciones de audio de las entrevistas se sometieron a un análisis de contenido. Las notas del diario de campo proporcionaron aportaciones adicionales al proceso de organización de las categorías, dándoles un contexto más preciso.

**Resultados.:**

Se constató que las participantes vivenciaban básicamente dos duelos, uno por la muerte del "familiar saludable" y otro por la "muerte anunciada" del mismo. Además, se comprobó que las participantes se enfrentaron a dos batallas principales: una para superar el cansancio - en sus vertientes objetiva y subjetiva - y otra para volverse plenamente aptas para la función que desempeñaban.

**Conclusión.:**

Las cuidadoras familiares de ancianos postrados en cama debido a enfermedades crónicas y degenerativas vivencian duelos y luchas que deben ser tenidos en cuenta desde el inicio del proceso de cuidado mediante acciones de salud mental dirigidas a sus necesidades.

## Resumo

**Objetivo.:**

Compreender lutos e lutas vivenciados por cuidadores(as) familiares de pessoas idosas acamadas devido a doenças crônico-degenerativas.

**Métodos.:**

O estudo possui caráter transversal e foi desenvolvido em consonância com as diretrizes do método clínico-qualitativo. A amostra foi constituída por 10 cuidadoras familiares de pessoas idosas acamadas devido a doenças crônico-degenerativas de uma cidade do interior do Estado de Minas Gerais, no Brasil. O fechamento amostral se deu por saturação. Os instrumentos utilizados foram um roteiro de entrevista semiestruturado e um diário de campo. As transcrições literais e integrais das gravações em áudio das entrevistas foram submetidas à análise de conteúdo. As anotações do diário de campo forneceram aportes adicionais para o processo de organização de categorias, conferindo-lhes um contexto mais acurado.

**Resultados.:**

Constatou-se que as participantes vivenciavam basicamente dois lutos, sendo um pela perda do “familiar saudável” e outro pela “morte anunciada” do mesmo. Ademais, verificou-se que as participantes enfrentavam duas batalhas principais: uma para superar o cansaço - em suas vertentes objetiva e subjetiva - e outra para se tornarem plenamente aptas para a função que desempenhavam.

**Conclusão.:**

As cuidadoras familiares de pessoas idosas acamadas devido a doenças crônico-degenerativas vivenciam lutos e lutas que devem ser levados em conta desde o início do processo de cuidado, por meio de ações de saúde mental dirigidas às suas necessidades.

## Introduction

The expression “family caregiver” refers to someone who provides unpaid and usually intuitive assistance to a sick family member, promoting his/her wellbeing.(1,2) Because this function is generally performed at home, living together is often necessary, especially when care is provided to elderly patients affected by chronic degenerative diseases and/or bedridden.(3) The reason is that the dependency and frailty of older individuals intensify in these conditions. On the other hand, cohabitation may lead family caregivers to neglect their own needs.(2)

Many studies worldwide have addressed the experiences of family caregivers from different perspectives, considering that a better understanding of this phenomenon can be instrumental in devising health actions at either the individual or collective level.(4-6) Most of these studies explore the grief triggered by the imminent death of patients in advanced stages of illness^,^(7,8) or by their deaths.(9,10) However, few studies address family caregivers during the care process, especially emphasizing anticipatory grief.(11) Hence, there is an important gap in the scientific literature, considering that grief encompasses a set of responses raised by a significant loss, not necessarily caused by death, and anticipatory grief is triggered by the progressive threat of loss.(12) Another evident gap is that studies seldom address coping strategies adopted by family caregivers to facilitate the care provided to bedridden elderly patients affected by chronic degenerative diseases.(13-15)

Even though the term “struggle” is not part of the technical terminology in the health field, family caregivers in many Brazilian regions - especially in the interior of Minas Gerais - use this term to refer to these coping strategies. So, it is associated with a wide range of care tasks that demand unconditional availability, including from an emotional point of view. Given the previous discussion, this study aims to understand grieves and struggles experienced by family caregivers providing care to bedridden elderly patients affected by chronic degenerative diseases. It is an excerpt of a larger study and was implemented *a posteriori* to highlight specific findings, which, in our view, required further investigation.

## Methods

This cross-sectional study was developed following the guidelines of the clinical-qualitative method, which is intended to clarify experiences that concern the health-disease-care *continuum* from the perspective of patients, family members, or health workers.(16) The sample was composed of 10 female family caregivers who met the following criteria: (1) were providing care for at least six months to a bedridden elderly patient with a chronic degenerative disease, and (2) were at least 18 years old.

It was not intentional to include only female family caregivers. Data saturation determined the sample size; thus, the recruitment of participants ceased when the authors identified that data were redundant and already met the study’s objectives.(17) The instruments used included a semi-structured interview and a field diary. Both are tools recurrently used in qualitative studies, and field diaries are usually adopted to facilitate access to data that complement information provided in the interviews.

Considering the objectives of the study from which this excerpt originates, the semi-structured interview was composed of 32 questions distributed into four axes. The first axis was composed of nine questions concerning how the family caregiver was chosen (e.g., “How was it determined who would be the family caregiver?”). The second axis included 10 questions addressing the care tasks (e.g., “What are your main responsibilities as a family caregiver?”). The third axis included 6 questions that investigated how the family caregiver performed his/her roles (e.g., “What are the difficulties and facilities you experience in the care process?”). Finally, the fourth axis encompassed 7 questions addressing the family caregivers’ self-care (e.g., “What do you do to take care of your own health?”).

A semi-structured interview script was used to encourage the participants to verbally express their feelings and thoughts regarding the topic under study.(18) A field diary was used by the primary author (a psychologist experienced with home visits and interviews who worked in a primary health care service at the time) to record her impressions regarding the participants’ contexts.(19) The participants were recruited from the list of a public home care service - predominantly aimed at elderly patients - located in a medium-sized city in the interior of Minas Gerais, Brazil. Those who met the inclusion criteria were invited by telephone to participate in the study. All the potential participants contacted were eligible, and none of them refused. Note that 30 individuals were listed; however, as the sample size was determined by saturation rather than exhaustion, we do not know how many of these individuals would be actually eligible. Therefore, we cannot accurately determine the number of individuals in the target population, considering that not all those assisted by the service were bedridden due to chronic degenerative diseases.

After the participants signed a free and informed consent form, the interviews were held in person, according to the participants’ convenient date and time, between October and December 2018. Considering the guidelines for clinical-qualitative studies, interviews preferably take place in the natural setting of care.(16) Hence, data collection was conducted in the homes where the participants performed their family caregiver role in all cases. Note that the author responsible for collecting data did not previously know any of the participants. Also, the participants chose the room they deemed to be the most appropriate to provide the interview and whether they wanted to be accompanied by other people or not.

These aspects were somewhat heterogeneous because participants 1 and 2 opted to be interviewed in the patients’ bedroom, while the remaining participants chose other rooms. Participants 3 and 4 chose to be accompanied by formal caregivers, participants 5 and 7 chose to be accompanied by their sisters, and participant 8 chose her granddaughter. There were no verbal or non-verbal expressions that embarrassed the participants. To reduce eventual biases, the companions also received clarification regarding the target population. Additionally, when the author responsible for data collecting was alone with the participants, she offered the possibility of an additional interview without any companions if the participants desired to review or detail any of the information provided. None of the participants requested an additional interview.

The interviews were audio-recorded and lasted 40 minutes on average, and only one interview was conducted with each of the participants. The author responsible for data collection took note in the field diary of observations and reflections regarding the participants’ non-verbal behavior and the home environment. The field diary was filled out immediately after the interviews to avoid potential embarrassment. Data collection was based on current ethical guidelines regulating research with human subjects in Brazil (Resolution CNS No. 466/2012). The study was approved by the Institutional Review Board at the university to which the authors are affiliated (Opinion Report CEP No. 2.798.042).

The *corpus* of analysis was mainly composed of verbatim transcriptions of the audio-recorded interviews. This material was submitted to content analysis, as proposed by Bardin.(20) According to Bardin, content analysis is intended to determine latent aspects of communications so that the process starts with fluctuating readings and culminates with the organization of categories that emerge from the coding process and result in significant results. Notes in the field diary provided additional contributions to the organization of categories, resulting in a more accurate context.

All the authors collaborated with the content analysis. The primary and fourth authors independently performed the fluctuating readings and organized preliminary categories based on relevance criterion and using the field diary as an additional resource. The preliminary categories were then refined, a process conducted by the primary and fourth authors throughout meetings. The second and third authors reviewed the categories and suggested some adjustments. After the primary and fourth authors reformulated specific aspects, the second and third authors reviewed the categories and validated them.

## Results

Most participants were married, presented a low level of education, and were the daughters of the elderly patient receiving care. Some disparities were found regarding the participants’ age (*M=*67.7 years) and how long they are providing care (*M=*8.3 years). This information is presented in Table 1. Note that participant 7 did not live with the elderly patient to whom she provides care, and only participant 9 reconciled her family caregiver role with a paid job, a job she sporadically performed outside the home. Most of the elderly patients were men (*n=*6) and presented complications and sequelae of neurological diseases, especially Alzheimer’s (*n=*6).

**Table 1:** Characterization of the participants according to age, marital status, education, kinship, and duration of care

Participants	Characteristics
1	55 years old / divorced / incomplete middle school / daughter / 10 years
2	82 years old / married / incomplete middle school / wife / 14 years
3	86 years old / married / incomplete middle school / wife / 10 years
4	86 years old / married / middle school / wife / 9 years
5	79 years old / widowed / college education / mother / 20 years
6	56 years old / single / middle school / daughter / 5 years
7	62 years old / married / middle school / daughter / 4 years
8	55 years old / divorced / incomplete college education / daughter / 3 years
9	54 years old / divorced / incomplete middle school / daughter / 7 years
10	62 years old / widowed / middle school / daughter / 1 year

Content analysis, according to the study’s objective, led to the emergence of two categories. The first category, called “Grieves,” sheds light on the participants’ experiences considering losses caused or accentuated at different spheres due to their family caregiver role. The second category, called “Struggles,” refers to the efforts required to overcome daily difficulties the participants had to face since they became family caregivers, considering repetitive and continuous care demands.

The first category revealed that the grief for the loss of a “healthy family member” was more prominent. From the participants’ perspectives, their elderly patient, after became ill, and especially after became bedridden, were no longer the same person. This grief process appears as a procedural phenomenon. It begins with the illness and intensifies with the emergence of complications and sequelae, negatively impacting the family dynamics. Note that this impact occurs at different levels, and changing social roles is only one of them.

One example illustrating this situation refers to one of the participants who improvised a rope system on the patient’s bed to allow him to sit up by himself. This system was noted in the field diary: “The patient sits there holding strings. As a statue, he seemed to sit on a swing that does not swing”. In Report 1, this participant explains how she rearranged the home environment, which suggests that care demands presented by a bedridden elderly patient may impose a need to reorganize the home, revealing transformations that occur in the context of affective relationships and social roles as a result of the loss of a “healthy family member”.

Report 1: *It’s been three years since I put these strings here. I looked at the window and thought: ‘if only I tied some strings, he’d be able to sit by himself’. So I cut some strings and tied them here. Then, he had difficulties getting on the bed. So I thought: ‘I’m going to put another string there’. Now, whenever he wants to sit, he uses the strings.* (Participant 2)

Another situation observed in the research field, in another participant’s home, support this line of reasoning, which led to the following observation recorded on the field diary: “The scene of illness is wide open at the house’s door. You can see from the street the bedridden patient in his bedroom. From the outside you can see that there is someone ill at home”. Report 2 refers to this situation and clarifies that the house was rearranged to confer more comfort to the patient. The patient started occupying one room at the house’s entrance, which used to be a living room. His wife’s bedroom remained in the same place, at the back of the house.

Report 2: *It used to be a living room [...], so we installed it [a larger window] there, arranged a single bed for him [...] and used that [a recently built access ramp] to take him outside.* (Participant 10) 

It is worth noting that, as showed by Reports 3, 4, and 5, the participants occasionally expressed grief for the patients’ progressive worsening clinical conditions, and as a consequence, sensed his/her finitude. Therefore, it seems reasonable to state that the results concerning the first category reveal that the participants also experienced grief for the “announced death”, though more discreetly, than the grief for the loss of a “healthy family member”. 

Report 3: *He [patient] is quiet, speechless, doesn’t talk, doesn’t walk [...] He doesn’t know anyone who enters there; he’s on oxygen* [...]. (Participant 3)

Report 4: *He [*patient*] used to swallow well, used to eat very well, now he doesn’t want anything.* (Participant 5) 

Report 5: *Because he still walked when he got sick. He was able to walk before he fell out of bed. So, it’s over for three years now.* (Participant 4)

The second category showed that the participants’ experiences as family caregivers were permeated by two significant struggles. One of which refers to the need to overcome the fatigue caused by their functions. The author responsible for data collection noted that most of the participants showed signs of exhaustion. The following entry in the field diary refers to a specific case: “During the interview, the participant kept her eyes sad and showed signs of tiredness”. Apparently, the participants’ fatigue resulted from two reasons. As shown by Reports 6, 7, and 8, one of which was an objective reason and concerned the care tasks under their responsibility.

Report 6:*You get really tired [...]. Sometimes you have to wake up in the middle of the night [to care for the patient]. I put my mobile to wake me up, so I go and take a look at her, you know, to see if she’s choking, you have to keep an eye on that. I sleep beside her. We sleep in the same room. I divorced my husband five years ago, and since then, I sleep with her*. (Participant 1)

Report 7*: In my free time, I just want to rest. I’m usually tired [for providing care to the patient] in my free time.* (Participant 2)

Report 8: *It’s tiresome, you know? [to provide care]. Sometimes, you spend the night without sleep a wink. It’s a struggle.* (Participant 6)

The second reason for the participants’ tiredness, as we inferred, was subjective and related to the complex and gradual process of reconstructing their personal and professional identities, determined by the complete abandonment of their previous social roles, with which they apparently became involved after they became family caregivers. They suggested that their lives were completely changed, and they needed to reinvent themselves after it. Reports 9, 10, and 11 are emblematic in this sense.

Report 9: *I didn’t live here, I lived abroad, in another city, another country. But then my mother needed me to come back, and I did. It was a radical change.* (Participant 9)

Report 10:*I was an assistant secretary. I got on leave in September, and it’s been two years. I got on leave to help and take care of my mother, I got on leave ahead of time, you know?*(Participant 7)

Report 11: *I used to do handcrafts, now I don’t anymore*... (Participant 8)

However, this process of identity reformulation has positive aspects as well, considering that most of the participants developed personal resources that started to be used to favor adaptation to the new context caused by the illness of the elderly patient to whom they provide care. As a consequence, they experienced a comforting sense of accomplishment. Some of them clearly stated that their role “is not easy” but “it’s gratifying”.

To conclude the second category, we point out that the participants apparently fought another fight, attempting to acquire the skills and competencies necessary to play their family caregiver role, mainly to deal with critical situations, as shown in Report 12. After all, these situations clearly revealed the participants’ limitations - mainly originated from a lack of technical training - regarding the performance of care tasks, consequently raising insecurities. However, as noted by Report 13, what made this struggle particularly challenging, at least for some of the participants, was a fear that the person receiving care would, at some point, require the use of intracorporeal devices.

Report 12: *We are not perfect [...] I do my best when she feels sick [...], but I feel insecure. Sometimes I freak out when she [patient] feels sick.* (Participant 6)

Report 13:*I’m afraid that she might need a tube because I have no practice. A breathing tube… Because they [physicians]* *say that it may get to this point, you know?*(Participant 1)

## Discussion

First, we need to clarify that the participants’ profile is in line with the profile of family caregivers reported by other studies conducted in Brazil concerning sex, kinship, and educational level.(21,22) Regarding the results more directly related to the study’s objective, note that the grief experienced for the loss of a “healthy family member” was the most evident among the participants. In some cases, this loss was revealed by the need to rearrange the home, which leads us to suggest that the grief related to the loss of a “healthy family member” may be synthesized by the loss of the place - concrete and symbolic - that these individuals previously occupied within the family.

In this sense, as defended by other authors,(22) the results highlight that relationships of reciprocity become relationships of dependence in the families in which the presence of a family caregiver is necessary. Perhaps this transformation is a facet of the grief for the loss of a “healthy family member”, and more subtly, of the grief for the patient’s “announced death”. After all, the death associated with chronic degenerative diseases usually occurs after long-term care provided at home and is preceded by many signs and symptoms.(12)

According to the participants’ reports, the grief for an “announced death” suggests anticipatory grief, because it concerns a situation in which death is very likely; however, one cannot estimate when it will actually occur.(12) This is a highly relevant finding, since some authors still doubt the possibility of anticipatory grief occur in family caregivers of patients who are not yet in advanced stage of illness.(23) Additionally, this grief expressed by the participants indicates that seeing the finitude of the elderly patient to whom they provide care caused them suffering. Equivalent findings are reported by previous studies,(5,24) revealing that considering one’s future at the long term triggers intense emotional mobilization among family caregivers.

This study also shows that the participants had to deal with the fatigue caused by the role they played in their routine lives, which, in our view, accrued from both the care provided*per se*, but also from the process of reconstructing their personal and professional identities; a process initiated when they became family caregivers. However, we note that this reconstruction process also entails a positive facet despite the burdensome negative aspects. The family caregivers composing other studies’ samples,(11,21) also reported specific beneficial changes in their lives resulting from providing care.

Furthermore, the participants endeavored to acquire the skills and competencies necessary to perform care tasks, representing part of their struggles. One integrative review(2) suggests that one factor that makes the transition to the family caregiver role so challenging is precisely the need to learn many procedures. However, the results found here concerning this specific aspect differed because they highlight the experiences of some of the participants who were afraid of being unable to provide care in the future for not being qualified to handle intracorporeal devices.

Therefore, the conclusion is that as family caregivers of bedridden elderly patients affected by chronic degenerative diseases, the participants experienced two types of grief, one for the loss of a “healthy family member” and another for the “announced death” of this patient. Note that the first grief stood out and encompassed the loss of the place the patient previously occupied in the family. Additionally, the participants also faced two main struggles: overcoming their objective and subjective fatigue and becoming skillful to perform their roles. These results support the practice of health workers as they reinforce the importance of implementing mental health actions for family caregivers since the beginning of the care process and highlight their need for a support network within and outside the family.

This study’s results also pose some questions that deserve to be further explored in the future. Specifically, multi-center, longitudinal studies are recommended to identify potential variations in how anticipatory grief is manifested during the care process. Note that this study has some limitations determined by its cross-sectional nature, in which a phenomenon was described - the experience of family caregivers performing their role within the home context - as spontaneously manifested at a given point in time. Another limitation refers to the fact that the participants were recruited from a single public home care service. 
